# Emerging Host, Emerging Parasites: Cardiopulmonary Nematodes in Italian Golden Jackals (*Canis aureus*)

**DOI:** 10.1155/tbed/9093298

**Published:** 2026-11-04

**Authors:** Elisabetta Ferraro, Lorenzo Frangini, Erica Marchiori, Giulia Simonato, Rudi Cassini, Stefano Pesaro, Paola Beraldo

**Affiliations:** ^1^ Department of Animal Medicine Production and Health University of Padova Legnaro Italy unipd.it; ^2^ Department of Agricultural Food Environmental and Animal Sciences University of Udine Udine Italy uniud.it

**Keywords:** *Angiostrongylus vasorum*, *Crenosoma vulpis*, *Dirofilaria immitis*, epidemiology, *Eucoleus aerophilus*, wildlife

## Abstract

Cardiopulmonary nematodes of canids are important veterinary pathogens, and their distribution across Europe is expanding, a trend driven by climate change and shifting wildlife dynamics. The golden jackal (*Canis aureus*), a highly adaptable mesocarnivore, could function as a key reservoir and intermediary host at the boundary between wild and domestic animals. This study provides a comprehensive, long‐term assessment of the cardiopulmonary nematode community in golden jackals from Northeastern Italy (Friuli Venezia Giulia [FVG]). From 2009 to 2025, 109 jackal carcasses were necropsied. The nematodes recovered were identified morphometrically and confirmed by PCR (targeting mtCOI, 18S rRNA and 12S rRNA genes). The infection parameters and the influence of host characteristics on infection probability were calculated. Spatial modelling was applied in order to assess how environmental and anthropogenic factors influence the probability of infection. Overall, 52 jackals (47.7%) were positive for at least one parasite species. The prevalence of *Angiostrongylus vasorum* was the highest (34.9%), followed by *Eucoleus aerophilus* (13.8%), *Crenosoma vulpis* (12.8%) and *Dirofilaria immitis* (4.6%). Host sex had a significant effect on the prevalence and abundance of *C. vulpis*, with higher rates in males. Mean annual precipitation and the extent of agricultural land cover (AGR) were significant positive predictors of *A. vasorum* and *E. aerophilus* infections. Our findings suggest that the golden jackal may serve as a reservoir for veterinary and zoonotic pathogens. As jackals increasingly exploit human‐dominated habitats, their role in maintaining and spreading these nematodes in the environment may become more relevant. Given the species’ ongoing westward expansion, integrated One Health surveillance programmes are essential to monitor pathogen spread within multispecies landscapes.

## 1. Introduction

Cardiopulmonary nematodes of canids have emerged as significant veterinary pathogens because of their broad host range and potential to cause severe, life‐threatening disease. In recent decades, climate change, altered vector ecology and shifting wildlife population dynamics have contributed to the geographic expansion of these parasites across Europe, with increasing reports in previously non‐endemic regions [1, 2]. This is particularly evident for the metastrongyloids *Angiostrongylus vasorum* and *Crenosoma vulpis*, the filarioid *Dirofilaria immitis* and the trichuroid *Eucoleus aerophilus* (syn. *Capillaria aerophila*) [2, 3].

*Angiostrongylus vasorum* (Nematoda: Angiostrongylidae) has an indirect life cycle involving gastropod intermediate hosts, with transmission also facilitated by paratenic hosts, such as amphibians and birds [4, 5]. Recognised as an emerging European pathogen [6], it primarily utilises red foxes (*Vulpes vulpes*) as its main reservoir [7], while domestic dogs and wild canids, including wolves (*Canis lupus*), golden jackals (*Canis aureus*) and red foxes, can develop significant pathological lesions, such as parasitic bronchopneumonia, lymphadenitis, meningoencephalitis and myocardial hypertrophy [8–11]. Its expanding geographic and host range is further evidenced by increasing documentation in wolves [11, 12], badgers (*Meles meles*) [13] and golden jackals [10, 14–17].

*Dirofilaria immitis* (Nematoda: Onchocercidae), transmitted exclusively by culicid mosquitoes (e.g., *Aedes* spp., *Anopheles* spp., *Culex* spp., *Mansonia* spp. and *Ochlerotatus* spp.), causes life‐threatening cardiopulmonary dirofilariosis in dogs and can infect a wide range of mammals, including humans, in whom microfilaremia is typically absent [18]. Wild canids, in contrast, sustain microfilaremia sufficient to infect vectors, making them serve as more consistent reservoir hosts, as demonstrated by the presence of microfilaremia, which enables vector infection [19].

*Eucoleus aerophilus* (Nematoda: Capillariidae) has a flexible life cycle, either direct or indirect via earthworms, and inhabits the tracheobronchial mucosa of various carnivores and, occasionally, humans [20, 21]. Its cosmopolitan distribution reflects its broad host range and environmentally resilient eggs [21]. *Crenosoma vulpis* (Nematoda: Crenosomidae) follows an indirect life cycle via gastropods and parasitises the trachea, bronchi and bronchioles of domestic and wild canids, mustelids and ursids [22, 23]. Clinical signs of both (chronic inflammatory pulmonary disorders) are well documented in domestic animals, but wildlife reports remain scarce and often non‐specific, risking misdiagnosis [1, 2].

Ecological conditions shape both the distribution and persistence of these nematodes. Warmer temperatures favour *A. vasorum* larval development in gastropods, whereas *C. vulpis* tolerates colder climates but depends on precipitation‐driven gastropod availability, which may limit its geographic distribution [3, 24, 25]. Although *E. aerophilus* is comparatively less dependent on climate because of its direct life cycle, it is still influenced by precipitation, as this affects egg survival and earthworm activity [25]. For vector‐borne *D. immitis*, warm and humid conditions favour mosquito reproduction and transmission [26].

Alongside environmental drivers, host ecology is equally critical: wild canids serve as key reservoirs, facilitating parasite persistence and dispersal [27]. In Italy, the golden jackal (*Canis aureus*) has emerged as a significant bridge host following its rapid westward expansion from the Balkans, with Friuli Venezia Giulia (FVG) as its core area since the mid‐1980s [28], acting as the primary transboundary ecological corridor linking Balkan populations to Western Europe and sustaining a continuous influx of dispersing individuals that can actively reshape the local epidemiological scenario. Recent evidence suggests that golden jackal spatial ecology is increasingly shaped by anthropogenic barriers and wolf recolonisation [29], with jackals favouring fragmented, human‐dominated landscapes, while wolves tend to occupy continuous, low‐disturbance forested Alpine areas [29, 30]. This occupancy of the wildlife–domestic animal–human interface may increase contact with parasites, vectors and infected hosts across both wild and domestic compartments, positioning the species as a potential reservoir and epidemiological bridge—including for zoonotic agents— underscoring the need for enhanced health surveillance.

In light of this evolving epidemiological scenario, the present study aimed to: (i) characterise the cardiopulmonary nematode fauna in Italian golden jackals from Northeastern Italy, building on previous evidence of *A. vasorum* and *D. immitis* co‐infection [10]; (ii) identify environmental drivers of their spatial distribution and (iii) contextualise these findings through a quantitative literature synthesis, with prevalence maps based on pooled estimates. By integrating long‐term surveillance data with ecological variables, this study provides a comprehensive framework for understanding the role of the golden jackal as a pathogen reservoir at the wildlife–domestic–human interface.

## 2. Materials and Methods

### 2.1. Study Area and Sample Collection

FVG (Northeastern Italy), bordering Slovenia and Austria, is an environmentally heterogeneous region spanning from the Po Valley lowlands (mean annual temperature 12–13°C) to the Alpine ranges (mean annual temperature <0°C), with regional precipitation consistently exceeding 1000 mm. The area hosts high mammalian diversity (>90 species), with widespread red fox and an expanding grey wolf population [31], whereas the Eurasian lynx (*Lynx lynx*) remains scarce [32].

For this study, golden jackal carcasses were collected between 2009 and 2025. Samples were obtained predominantly from road‐killed individuals via the InfoFaunaFVG regional wildlife health surveillance network [33]. Because the study was based exclusively on post‐mortem examinations of animals found dead, no ethical approval was required.

### 2.2. Necropsy and Parasitological Analyses

Prior to necropsy, sex and morphometric measurements were recorded of each individual, and age category (adult, subadult and pup) was determined based on dental development and tooth eruption patterns. Upon opening the thoracic cavity, a systematic gross examination was performed. The heart, lungs, pulmonary vessels and tracheobronchial tree were examined through direct dissection and/or under a stereomicroscope, and lung tissue was additionally processed using a sedimentation technique (modified from Arcenillas‐Hernández et al. [1]). All recovered parasites were counted and preserved in saline solution until taxonomic identification was performed using established morphometric keys [22, 34–36].

### 2.3. Molecular Identification

Morphological identification was confirmed by molecular analysis, selecting at least one parasitic specimen of each putative species from different individual hosts following morphological observation. Helminths were removed from 70% ethanol, washed in phosphate‐buffered saline and left to dry overnight. DNA was extracted using the NucleoSpin Tissue Kit (Macherey‐Nagel, Düren, Germany) according to the manufacturer’s instructions. PCR amplification was performed using different primer sets chosen according to the helminth group. For *A. vasorum*, *E. aerophilus* and *C. vulpis*, amplification targeted the 18S rRNA (965F/1573R, [37]) and the mtCOI (LCO‐1490/HCO‐2198, [38]) genes. For *D. immitis*, PCR amplification targeted the 12S rRNA (Diro12S‐F/Diro12S‐R) and mtCOI (Diro‐cox1‐F/Diro‐cox1‐R) genes, following the protocol of Suzuki et al. [39]. PCR products were visualised on a 2% agarose gel, and the amplicons were purified with ExoSAP (Thermo Fisher Scientific Inc., USA) and sequenced in both directions (Macrogen Europe, The Netherlands). The resulting nucleotide sequences from the recovered helminth groups were assembled using Unipro UGENE v53.1 software [40] and compared with homologous sequences deposited in the NCBI GenBank database through the BLAST tool (https://blast.ncbi.nlm.nih.gov/Blast.cgi).

### 2.4. Environmental Variables

To assess the impact of landscape features on the probability of parasite infection, a suite of environmental and anthropogenic variables was extracted as potential predictors of the parasites’ spatial distribution. These variables served as proxies for the presence and abundance of intermediate hosts, specifically gastropods (*Gastropoda* spp.) [41] and amphibians (*Amphibia* spp.) [4], as well as for sympatric definitive hosts, such as domestic dogs and red foxes, which may amplify the abundance of infective forms.

To characterise the ecological requirements of intermediate hosts, the following covariates were employed: elevation (DEM), yearly average temperature (YAT), yearly average precipitation (YAP), agricultural land cover (AGR) and surface water coverage (WET). To account for the role of definitive hosts in parasite maintenance, the human footprint index (HFI) [42] and the density of road‐killed foxes (FOX) were used as proxies for dog and fox presence, respectively. The FOX variable was calculated via kernel density estimation (KDE) [33], using a 740 m radius, representing the average home range size for red foxes in Europe [43].

All landscape and climatic metrics were summarised within a 26.95 km^2^ buffer centring on each sampling point, corresponding to the average home range of golden jackals in the study area (Filacorda, unpublished results). For each buffer, the mean values of DEM, HFI, FOX, YAT and YAP were calculated, along with the percentage cover of AGR and WET. DEM and WET data were sourced from the Copernicus Land Monitoring Service, while AGR was obtained from Marsoner et al. [44]. Climatic data (YAT and YAP) were obtained from regional weather monitoring datasets [45]. All geospatial calculations were performed using the R packages ‘terra’ [46] and ‘landscapemetrics’ [47].

### 2.5. Statistical Analysis

#### 2.5.1. Descriptive Statistics and Host‐Related Factors

For each parasite species, prevalence with 95% confidence intervals (Clopper–Pearson method), mean intensity, mean and relative abundance and the aggregation parameter (*k*) were calculated. The influence of host‐related variables (age and sex) on parasite positivity was assessed using Pearson’s chi‐squared test and Fisher’s exact test, while their effect on the parasite burden (abundance) was evaluated using the Kruskal–Wallis test. Statistical significance was set at *p* < 0.05.

#### 2.5.2. Data Pre‐Processing and Multicollinearity

To ensure the robustness of the environmental models, the dataset was refined to address biological and statistical biases. First, road‐killed subadults collected between January and May were excluded to minimise the influence of dispersing individuals on local prevalence estimates [48]. Second, nine individuals identified as outliers for the surface water covariate (WET) were removed. Multicollinearity among the remaining environmental and anthropogenic variables was assessed using the variance inflation factor (VIF). Variables were removed iteratively until all remaining predictors showed a VIF < 3 [49]; consequently, YAT and DEM were excluded from the models.

#### 2.5.3. Spatial Modelling and Model Selection

The probability of parasite infection was initially assessed using generalised linear mixed models (GLMMs) with ‘year’ as a random effect. However, as the random effect accounted for zero variance, the analysis proceeded with generalised linear models (GLMs) with a binomial distribution. Spatial autocorrelation in the model residuals was assessed using Moran’s I test via the ’DHARMa’ R package [50]. When significant spatial autocorrelation was detected, a spatial GLMM (spGLMM) with a Matérn random intercept was employed using the ‘spaMM’ R package [51].

Model selection was performed by testing multiple combinations of predictors, which were ranked by Akaike’s information criterion (AIC) using the ‘MuMIn’ R package [52]. Competing models were identified using ΔAIC < 2 [53]. Final model assumptions were verified using residual diagnostics in ‘DHARMa’ [50], and marginal effects of significant predictors were visualised with the ‘ggeffect’ R package [54]. All statistical analyses were conducted in R ([55], version 4.3.2).

### 2.6. Literature Search and Spatial Visualisation of Cardiopulmonary Nematode Prevalence

A systematic literature review was conducted to complement our findings with existing data generated from similar epidemiological surveys, focusing exclusively on necropsy‐based studies in Europe (geographic Europe). The PubMed and Web of Science databases were searched using combinations of the following terms: ‘jackals’ AND *‘angiostrongylus vasorum*’, *‘crenosoma vulpis*’, *‘dirofilaria immitis*’ and *‘eucoleus aerophilus*’ OR *‘capillaria aerophila*’. Studies were included only if they explicitly reported both the total number of golden jackals and the number of positive individuals. When multiple studies were available for the same country or region, a pooled prevalence with a 95% confidence interval was calculated using a random‐effects model (DerSimonian‐Laird, logit transformation). This analysis was implemented using the R package ‘meta’ (function ‘metaprop’) [56], and statistical heterogeneity was quantified using *I* ^2^ and Cochran’s Q test.

The geographical distribution of parasite prevalence across Europe was visualised in QGIS [57]. For each study, prevalence data were assigned to the corresponding administrative unit (e.g., county) or, where precise coordinates were provided, mapped as point data. Studies reporting aggregated data without regional breakdowns were represented by a single merged polygon covering the entire study area. To avoid displaying prevalence estimates derived from critically small sample sizes, administrative units with fewer than five examined golden jackals were pooled with neighbouring units from the same study. Spatial boundaries were obtained from OpenStreetMap (https://www.openstreetmap.org/) and geoBoundaries [58], and all layers were projected to the EPSG:4326 coordinate reference system.

## 3. Results

### 3.1. Parasitological and Anatomopathological Findings

A total of 109 golden jackals were examined for cardiopulmonary nematodes. The temporal distribution of the samples was skewed towards the latter part of the study period: 20 individuals were collected between 2009 and 2021, while 89 (81.6%) were obtained between 2021 and 2025. This trend reflects the significant population growth and range expansion of this wild canid within the region over the past decade. The study population consisted of 75 adults (68.8%), 33 subadults (30.3%) and one pup (0.9%) (grouped with subadults for statistical analyses), with a sex ratio of 1.3:1 (62 females and 47 males).

Overall, 52 jackals (47.7%; 95% CI: 38.6–57.0) were positive for at least one nematode species. A total of 484 helminths were recovered and identified under the applied dissection protocol. Morphological observation allowed them to be ascribed to four species: *E. aerophilus*, *C. vulpis*, *A. vasorum* and *D. immitis*. Additionally, a single *D. repens* specimen was retrieved from the right cardiac ventricle of one individual co‐infected with *A. vasorum*; this erratic localisation has been documented elsewhere [59] and was not included in subsequent analyses. The distribution of positive animals is shown in Figure 1 for each of the four species.

**Figure 1:**
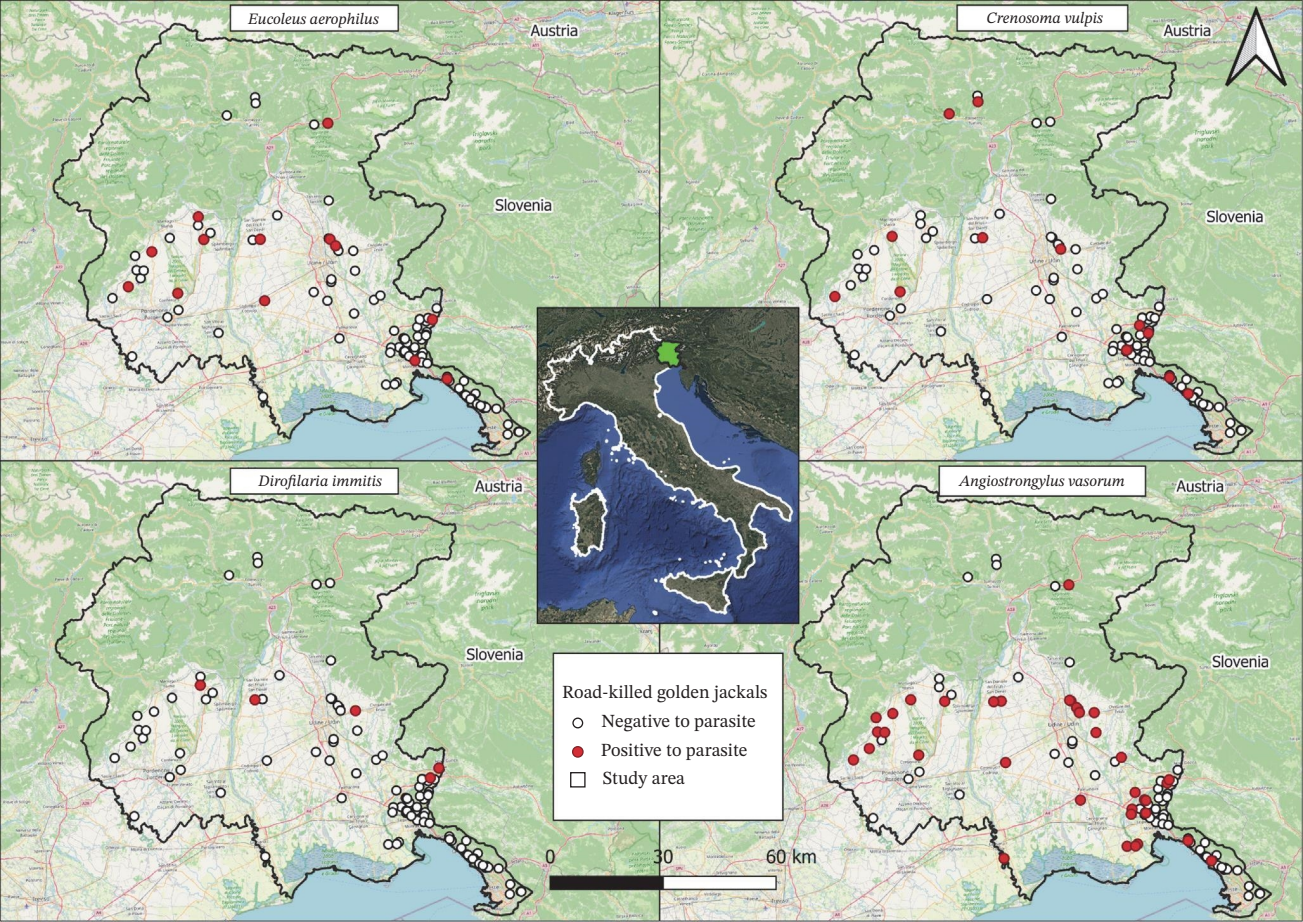
Distribution of examined golden jackals and their positivity to the four investigated parasites. Each panel represents the positivity (red dots) to the infection by the considered parasite. *Source*: map: OpenStreetMap, https://www.openstreetmap.org/.

*Angiostrongylus vasorum* was the most prevalent species (34.9%), followed by *E. aerophilus* (13.8%), *C. vulpis* (12.8%) and *D. immitis* (4.6%). Table 1 presents the detailed quantitative descriptors for each parasite. All species showed an aggregated distribution (*k* < 1). Notably, *A. vasorum* larvae were also recovered from lung tissue washings, confirming successful reproduction within this host.

**Table 1:** Parameters of infection of cardiopulmonary nematodes in golden jackals (*n* = 109).

Parasitological parameter	*A. vasorum*	*C. vulpis*	*D. immitis*	*E. aerophilus*
Positive individuals	38	14	5	15
Prevalence % (95% CI)	34.9 (26.0–44.6)	12.8 (7.2–20.6)	4.6 (1.5–10.4)	13.8 (7.9–21.7)
Parasites recovered	382	65	16	21
Range	1–70	1–24	1–6	1–4
Mean abundance (±SE)	3.5 (±1.0)	0.6 (±0.3)	0.1 (±0.1)	0.2 (±0.1)
Relative abundance (%)	79.1	13.5	3.1	4.1
Mean intensity (±SE)	10.1 (±2.7)	4.6 (±1.8)	3.2 (±0.8)	1.4 (±0.2)
Aggregation (*k*)	0.1249	0.0551	0.0242	0.2808

Abbreviation: SE, standard error.

Regarding pathological findings, significant gross lesions were observed exclusively in association with *A. vasorum*. In individuals with a burden of at least five parasites (*n* = 18/38, 39.5%), the lungs displayed a characteristic marbled appearance (Figure 2). The pulmonary parenchyma exhibited severe, multifocal‐to‐coalescing, dark purple to red‐brown areas of consolidation (consistent with granulomatous and haemorrhagic pneumonia), affecting roughly 50% of the visible surface. These consolidated regions alternated with pale pink, emphysematous tissue, with a distinct predilection for the lobar margins and dorsocaudal aspects. Notably, adults of *A. vasorum* were also recovered from smaller pulmonary vessels. Histopathological analysis confirmed severe multifocal granulomatous interstitial pneumonia.

**Figure 2:**
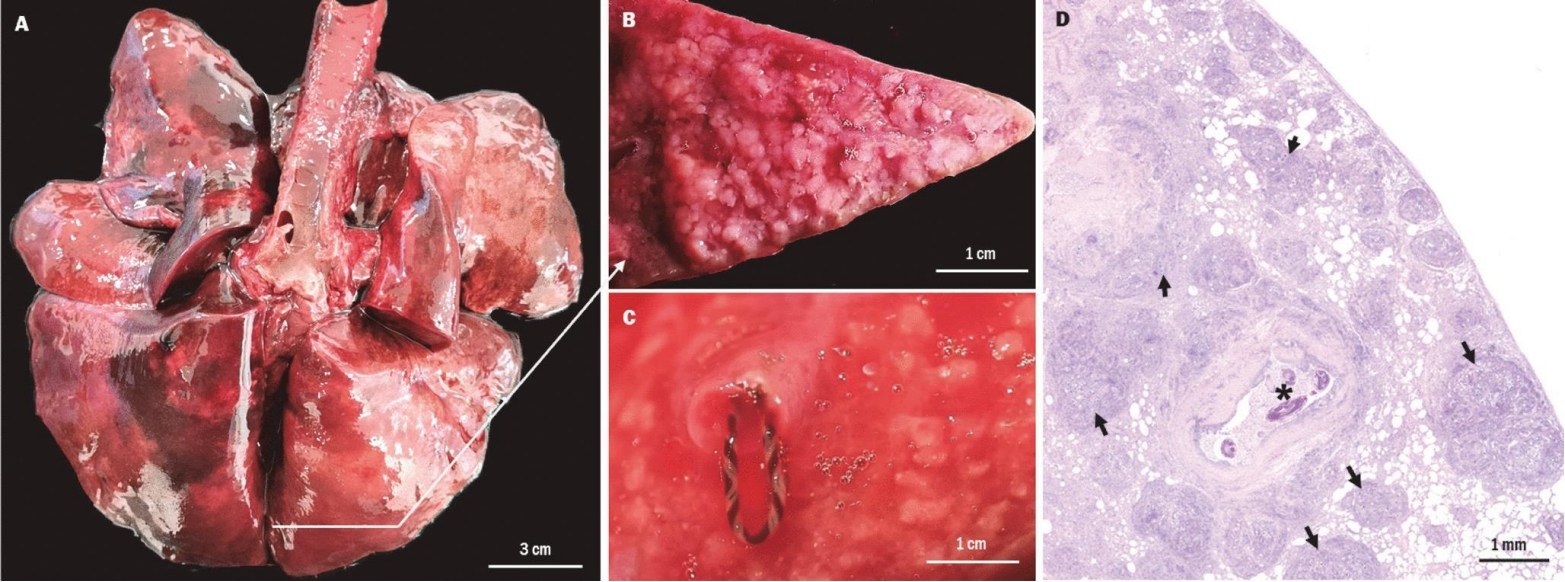
Anatomopathological findings in the case of *A. vasorum* infection. (A) Gross findings of the golden jackal lungs, which showed a firm consistency with multifocal consolidated areas. (B) Portion of the caudal lung lobe parenchyma displayed a severe, diffuse granular texture, characterised by the presence of many small, firm and subpleural nodular granulomas. (C) Adult female of *A. vasorum* within a pulmonary artery. (D) Lung parenchyma shows multifocal granulomatous interstitial pneumonia; an adult *A. vasorum* is visible in a pulmonary artery (asterisk), embryonated eggs and first‐stage larvae are embedded within the inflammatory foci (arrows).

Co‐infection patterns were common (Figure 3): 33 jackals infected (63.5%) harboured a single species, 17 (32.7%) exhibited double infections and two (3.8%) were positive for three different nematodes. Of note, *D. immitis* was found exclusively in co‐infection with *A. vasorum*. One animal, not shown in Figure 3, was co‐infected with *A. vasorum* and *D. repens*.

**Figure 3:**
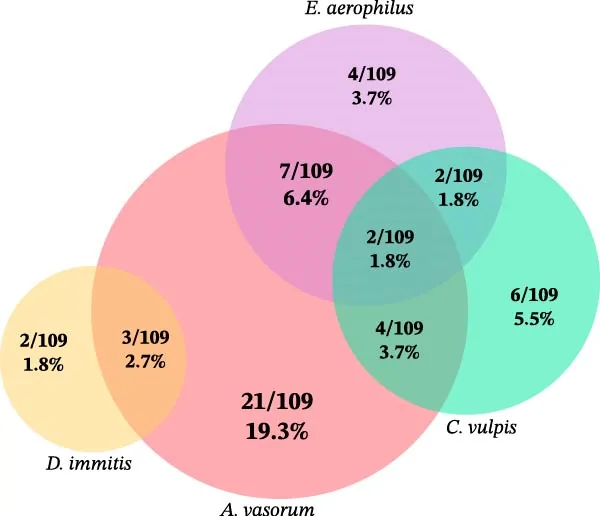
Single and multiple infections for the cardiopulmonary nematodes recovered in golden jackals.

### 3.2. Molecular Confirmation of Recovered Nematodes

Molecular results should be interpreted as confirmatory of morphological identification rather than representative of the genetic diversity within each parasite species. Good‐quality 18S sequences were obtained from specimens of *A. vasorum* (*n* = 2, from two golden jackals), *E. aerophilus* (*n* = 1, from one golden jackal) and *C. vulpis* (*n* = 1, from one golden jackal). Alignment of the two *A. vasorum* sequences showed that they were identical. The 18S sequences showed high sequence identity (98.5%–100%) over their respective alignment lengths (501–569 bp, 517 bp, and 571 bp, respectively) with reference strains available in GenBank (EF514916.1, KX962356.1 and KR920038.1, respectively).

Subsequent analysis of the mtCOI gene yielded two good‐quality sequences for *A. vasorum* (510–520 bp) and one for *C. vulpis* (220 bp). The *A. vasorum* mtCOI sequences showed a single nucleotide polymorphism at position 420 bp. Compared with GenBank reference sequences (NC_018602.1 and PP106440.1, respectively), the mtCOI sequences showed slightly lower identity (96.8%–99.6%). No mtCOI sequence was obtained for *E. aerophilus*.

For *D. immitis* (*n* = 3, from three golden jackals), amplification of the 12S rRNA gene yielded three good‐quality sequences (400 bp). The three identical sequences showed high identity (99.5%–100%) with the GenBank reference strain MZ678897.1. Amplification of the mtCOI gene likewise yielded three identical, good‐quality sequences (600 bp), showing high identity (99.5%–100%) with reference sequences for *D. immitis* (PV524386.1 and MW577348.1).

All newly generated sequences were deposited in GenBank (PZ748145, PZ748146, PZ748245, PZ748248‐50, PZ748255‐57 and PZ748275‐78).

### 3.3. Host Drivers of Parasite Positivity and Abundance

Host age did not significantly affect the parasite prevalence or abundance. By contrast, host sex was a significant predictor of *C. vulpis*: both the probability of infection (*p* = 0.0016) and mean parasite abundance (*p* = 0.0007) were significantly higher in males than in females (Table 2).

**Table 2:** Number of golden jackals examined, divided by age, sex, per each parasite recovered.

Category		*N*	*A. vasorum*		*C. vulpis*		*D. immitis*		*E. aerophilus*	
			Positive (%) [95% CI]	MA ± SE	Positive (%) [95% CI]	MA ± SE	Positive (%) [95% CI]	MA ± SE	Positive (%) [95% CI]	MA ± SE
Age	Adult	75	29 (38.8) [27.6–50.6]	4.7 ± 1.5	7 (9.3) [3.8–18.3]	0.4 ± 0.2	4 (5.3) [1.5–13.1]	0.2 ± 0.1	10 (13.3) [6.6–23.1]	0.1 ± 0.0
	Subadult	34	9 (26.5) [12.9–44.3]	0.8 ± 0.3	7 (20.6) [8.7–37.9]	1.1 ± 0.7	1 (2.9) [0.1–15.3]	0.1 ± 0.1	5 (14.7) [4.9–31.1]	0.3 ± 0.1

Sex	Female	62	20 (32.2) [20.9–45.3]	3.0 ± 1.2	**2 (3.2)** **[0.4–11.2]**	**0.1 ± 0.0**	3 (4.8) [1.0–13.5]	0.1 ± 0.1	10 (16.1) [8.0–27.7]	0.3 ± 0.1
	Male	47	18 (38.3) [24.5–53.6]	4.2 ± 1.8	**12 (25.5)** **[13.9–40.3]**	**1.3 ± 0.6**	2 (4.3) [0.5–14.5]	0.2 ± 0.1	5 (10.6) [3.5–23.1]	0.1 ± 0.0

*Note:* Lines in bold highlight statistically significant differences (*p* < 0.05).

Abbreviation: MA ± SE, mean abundance ± standard error.

### 3.4. Environmental Drivers of Parasite Positivity

After excluding dispersing subadults and outliers, the final modelling dataset comprised 92 jackals. *Dirofilaria immitis* was excluded from the spatial analysis due to its low prevalence (~5%). Models for *E. aerophilus* and *A. vasorum* showed no residual autocorrelation, allowing us to perform GLMs, whereas a spGLMM was employed for *C. vulpis*. Among the 32 model combinations tested, four models were considered competitors (i.e., ΔAIC < 2) for *E. aerophilus* and three for *A. vasorum* (Table 3). For both parasites, YAP and AGR were statistically significant and positively associated with the probability of being positive (Figure 4). Conversely, no significant environmental associations were detected for *C. vulpis*.

**Figure 4:**
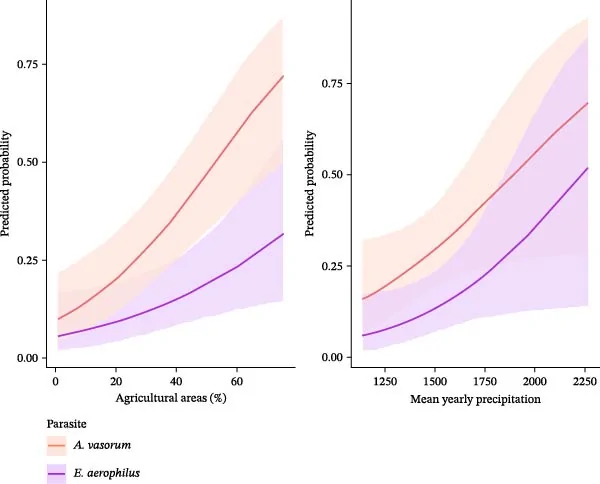
Marginal effect of significant variables on being positive for *E. aerophilus* and *A. vasorum*. The percentage of agricultural areas and yearly average precipitation were positively associated with being positive for the parasites.

**Table 3:** Generalised linear models (GLMs) ranking, with the best model in italics.

Variables	df	logLik	AICc	Δ AICc	Weight
*E. aerophilus*					
* YAP* ^∗^+*AGR* ^∗^	3	−33.971	74.215	0.000	0.208
HFI + AGR ^∗^	3	−34.664	75.601	1.386	0.104
AGR ^∗^	2	−35.819	75.772	1.557	0.095
YAP + HFI + AGR ^∗^	4	−33.783	76.026	1.811	0.084
*A. vasorum*					
* YAP* ^∗^ *+AGR* ^∗^	3	−43.853	93.978	0.000	0.278
AGR ^∗^	2	−45.774	95.683	1.705	0.119
YAP ^∗^+HFI + AGR ^∗^	4	−43.674	95.807	1.829	0.112

*Note:* Variables statistically significant (i.e. *p* < 0.05) are denoted by ‘ ^∗^’. ΔAICc, the difference with the AICc value of the best model; AGR, percentage of agricultural areas.

Abbreviations: AICc, corrected Akaike’s information criterion corrected; df, degrees of freedom; HFI, human footprint index; logLik, log likelihood; YAP, yearly average precipitation.

### 3.5. Literature Search and Spatial Visualisation

Based on the literature search, 16 studies investigating cardiopulmonary nematodes in golden jackals in Europe were identified. Three were excluded because prevalence was assessed using molecular methods rather than direct necropsy examination, precluding direct comparability with the remaining studies. One additional study was excluded for insufficient reporting of the number of positive animals. A total of 12 studies were therefore retained and used to generate the prevalence maps (Figure 5). For Romania, where two independent studies were available, pooled analysis (random‐effects model, logit transformation) yielded a combined *D. immitis* prevalence of 18.8% (95% CI: 12.7%–26.9%) in golden jackals, with no evidence of heterogeneity between studies (*I* ^2^ = 0%, *Q* = 0.01, *p* = 0.94).

**Figure 5:**
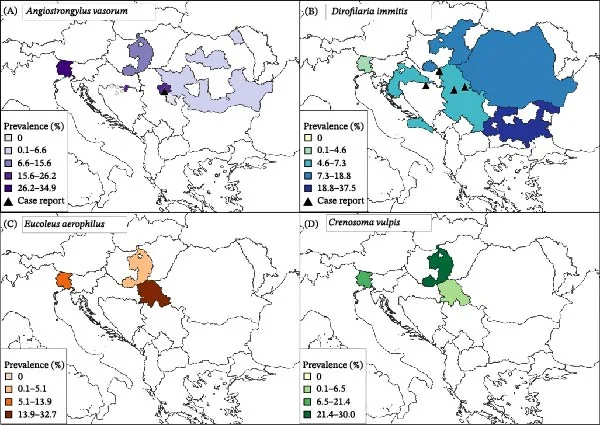
Spatial visualisation of cardiopulmonary nematode prevalence in golden jackals across Europe, based on published necropsy‐based studies. (A) Prevalence values and case report of *Angiostrongylus vasorum*. *Sources:* [14–17]. (B) Prevalence values and case reports of *Dirofilaria immitis*. *Sources:* [8, 16, 60–66]. (C) Prevalence values of *Eucoleus aerophilus*. *Sources:* [17, 67]. (D) Prevalence values of *Crenosoma vulpis*. *Sources:* [17, 67]. Spatial boundaries were obtained from OpenStreetMap (https://www.openstreetmap.org/) and geoBoundaries [58].

## 4. Discussion

This study provides a comprehensive assessment of cardiopulmonary nematodes in golden jackals from Northeastern Italy (FVG region) over 16 years, confirming the species’ role as a key host in the epidemiology of these parasites. As golden jackals expand across the continent, their potential to act as bridge hosts and wild reservoirs for zoonotic and veterinary pathogens requires integrated surveillance, particularly at the wildlife–domestic interface [27].

Our survey identified the cardiopulmonary nematodes most frequently found in other European canids. Even though *Oslerus osleri* and *Filaroides hirti* are sometimes recorded in certain populations of foxes and wolves in Europe, they were not found in our sample of jackals. This finding is consistent with the fact that these species have not previously been reported in golden jackals, even though they are occasionally present in other Italian wild canids and in domestic dogs [68, 69].

*Angiostrongylus vasorum* was the most prevalent species (34.9%) and was associated with the most severe pathological findings. While clinical disease, such as verminous pneumonia and right‐sided myocardial thickening, is well documented in dogs and red foxes [6], *A. vasorum* infections can also cause life‐threatening complications in jackals [10]. The frequency of co‐infections further complicates this epidemiological picture: *A. vasorum*‐positive jackals often harboured *C. vulpis* and *E. aerophilus* simultaneously, a pattern also frequently reported in red foxes [21]. The pathological impact of such polyparasitism is likely additive as these nematodes occupy distinct anatomical niches (*E. aerophilus* in the large bronchi, *C. vulpis* in the smaller airways, and *A. vasorum* in the pulmonary arteries), potentially severely compromising host fitness [21].

In Europe, *A. vasorum* has a fragmented distribution with localised endemic foci [3]. Our findings suggest a recent expansion into FVG, where autochthonous cases were undetected before 2018 [70–71]. However, recent dog seroprevalence has reached 7.5%, indicating that the parasite is becoming established [72]. In this context, the role of the red fox in sustaining and amplifying local transmission warrants particular attention. Red foxes lack protective immunity against *A. vasorum* and continuously shed larvae, leading to environmental parasite accumulation [7]. Notably, the recovery of larvae from lung washings confirms that the golden jackal is a highly competent definitive host rather than a mere spillover species. In FVG, transmission could be amplified by the sympatric overlap of these two competent canids. This is further supported, although not statistically significant, by the higher prevalence and mean abundance in adults compared to subadults, suggesting cumulative environmental exposure over time.

The prevalence of *A. vasorum* recorded in the present study (34.9%) is remarkably higher than those reported across Eastern Europe, where values rarely exceed 10% in countries such as Bosnia‐Herzegovina, Hungary, Romania and Serbia [14–17]. While a localised peak of 24% was recorded in South Banat, Serbia [14], the high rate found in FVG suggests favourable local conditions for transmission. It could be argued that our reliance on primarily road‐killed individuals might introduce a sampling bias; heavily parasitised jackals with compromised fitness may be more susceptible to vehicular collisions, thereby inflating the prevalence estimates compared to hunted or culled populations. However, current evidence from other European regions suggests that sampling methods (road‐killed vs. culled) do not significantly skew these findings as studies on hunted jackals have reported similarly high parasitic pressures [14]. Thus, our data likely provide a representative estimate of the actual infection levels in the FVG jackal population.

*Dirofilaria immitis* exhibited the lowest prevalence (4.6%) among the cardiopulmonary nematodes detected in this study. Despite this relatively low occurrence, *D. immitis* remains a central focus of research in European jackals, reflecting its significant pathological impact on both wildlife and domestic animal health. In golden jackals, prevalence shows a distinct east‐to‐west decline across Europe: reported values range from peaks in Bulgaria (37.5%) and Romania (~19%) to intermediate or lower levels (3.3%–7.6%) throughout the Balkan Peninsula [16, 60–63, 65, 66, 73]. The 4.6% prevalence observed in FVG jackals aligns with the lower end of this geographic trend and closely mirrors the recent prevalence reported in wolves from Central–Northern Italy (3.5%) [74], suggesting comparable exposure levels among sympatric wild canids. The active circulation of *D. immitis* in FVG is further corroborated by incidental reports in domestic and wild felines [75].

The epidemiological context of *D. immitis* in Northeastern Italy must be interpreted within the historical framework of the Po River Valley, one of Europe’s most hyperendemic areas for canine dirofilariosis [76]. While enhanced veterinary awareness and the widespread use of prophylaxis have successfully reduced the prevalence in domestic dogs, the parasite persists in untreated populations [77]. Climate change projections further complicate this scenario: habitat suitability models for *Aedes albopictus* and *Culex pipiens* identify the Po River Valley as a persistent high‐risk zone. Elevated temperatures, together with extensive natural and artificial water networks, promote vector proliferation, while high human density exacerbates domestic transmission cycles [78]. In this context, expanding wild canid populations, characterised by high dispersal capabilities, may act as another susceptible host, potentially maintaining the infection in the ecosystem.

The detection of *E. aerophilus* at an intermediate prevalence (13.8%) is consistent with its widespread occurrence in European wild canids. Despite its cosmopolitan distribution, reports on golden jackals have historically been limited to Hungary and Serbia [17, 67]. Our findings suggest that this expanding canid has been largely overlooked in prior surveys rather than being refractory to infection. As a competent host that frequently inhabits anthropised landscapes, the golden jackal poses a potential risk of environmental egg contamination, particularly in fragmented habitats where the wildlife–human interface is most pronounced.

Similarly, *C. vulpis* was detected at a prevalence of 12.8%. Consistent with findings in red foxes [79, 80], our jackal sample exhibited a significant male bias in infection. Since this bias was absent for other surveyed nematodes, a general immunosuppressive mechanism is unlikely; instead, this pattern is more parsimoniously explained by behavioural differences. Male golden jackals maintain larger home ranges [81], which likely increases their exposure to moist, densely vegetated microhabitats that support high densities of gastropod intermediate hosts. Published records of *C. vulpis* in jackals remain scarce [17, 67], and our data reinforce the role of this expanding carnivore as a highly competent host for this lungworm.

The GLMs identified YAP and AGR as significant positive predictors of *A. vasorum* and *E. aerophilus* infections. Conversely, no significant environmental associations were detected for *C. vulpis*. These findings are broadly consistent with the established eco‐epidemiology of these nematodes and offer preliminary insight into the landscape‐level factors governing their spatial distribution in Northeastern Italy. However, given the relatively low prevalence observed and the resulting limited statistical power, these associations should be regarded as tentative rather than confirmatory. The positive correlation between *A. vasorum* prevalence and rainfall is fundamentally driven by intermediate host ecology as elevated humidity favours the survival and activity of competent terrestrial gastropods. In endemic regions, larvae have been frequently recovered from various slugs and snails, such as *Cornu aspersum* (syn. *Helix aspersa*), *Arion* spp. (like *A. vulgaris*, *A. ater*, *A. hortensis*) and *Deroceras reticulatum* [82–83]. Cultivated landscapes may further optimise these transmission cycles through artificial irrigation and ‘edge effects’, which create suitable microclimatic niches while supporting high red‐fox densities [84]. This shared ecological requirement explains why *E. aerophilus* exhibited identical environmental associations as moisture‐rich environments also facilitate the persistence of its eggs and the availability of earthworm paratenic hosts.

The lack of significant environmental predictors for *C. vulpis* likely reflects its broad ecological tolerance [24] and resilience to low temperatures. This differential response aligns with observations that *A. vasorum* is climatically constrained to specific lowland areas [25], whereas *C. vulpis* maintains a more widespread distribution across diverse moisture gradients. Consequently, the transmission drivers of *C. vulpis* in FVG may operate at finer spatial scales or depend on microclimatic variables that were not captured by the macro‐environmental predictors used in our study.

Such geographic discrepancies, including weak or contrasting environmental associations reported in other studies [85], highlight the challenge of modelling parasites with patchy or focal distributions. When regional prevalence is low, statistical models may reflect the spatial distribution of a few positive cases rather than true ecological gradients. Therefore, our environmental outputs should be interpreted as exploratory, emphasising the need for targeted, small‐scale epidemiological surveillance to better define the risk at the wildlife–domestic interface.

## 5. Conclusion

This study provides the first comprehensive, long‐term characterisation of the cardiopulmonary parasite community in the golden jackal in Northeastern Italy, offering preliminary evidence that this expanding canid may act as a reservoir and bridge host for several parasites of veterinary and zoonotic importance. Our findings suggest that the spatial distribution of these parasites may be influenced by environmental and anthropogenic drivers, with YAP and AGR potentially facilitating the transmission cycles of *A. vasorum* and *E. aerophilus*, although these associations should be interpreted with caution given the relatively low prevalence observed and the resulting limited statistical power of the model. As jackals increasingly exploit fragmented, human‐dominated landscapes and partly avoid competition with recolonising wolves, their role at the wildlife–domestic interface could become more relevant, potentially heightening the risk of parasite spillover to domestic dogs.

Furthermore, the documentation of frequent co‐infections and the presence of zoonotic species, such as *D. immitis* and *E. aerophilus*, points to a potential role of this canid in the environmental maintenance and dispersal of these nematodes, although further studies are needed to clarify the extent of this contribution. Given the ongoing westward expansion of the golden jackal across Europe, the implementation of One Health surveillance programmes is essential to monitor pathogen spread and to develop effective management strategies in increasingly multispecies landscapes.

## Author Contributions

**Elisabetta Ferraro:** conceptualisation, data curation, formal analysis, investigation, visualisation, writing – original draft preparation, writing – review and editing. **Lorenzo Frangini**: formal analysis, visualisation, writing – original draft preparation, writing – review and editing. **Erica Marchiori**: investigation, resources, writing – review and editing. **Giulia Simonato**: resources, writing – review and editing. **Rudi Cassini**: resources, supervision, writing – review and editing. **Stefano Pesaro**: investigation, writing – review and editing. **Paola Beraldo**: conceptualisation, data curation, formal analysis, investigation, resources, supervision, writing – original draft preparation, writing – review and editing.

## Funding

This research did not receive any specific grant from funding agencies in the public, commercial or not‐for‐profit sectors.

## Disclosure

The authors have nothing to report.

## Ethics Statement

This study did not involve live animals or human participants. It was based exclusively on the post‐mortem examination of animals found dead as part of regional wildlife health monitoring. Therefore, no ethical approval was required.

## Conflicts of Interest

The authors declare no conflicts of interest.

## Data Availability

The data that support the findings of this study are openly available in Mendeley Data at DOI: 10.17632/r3ff2p87f9.1.
